# Post-operative radiographic measures of pelvic limb alignment in dogs with medial patellar luxation after trochlear wedge recession versus trochlear block recession surgery

**DOI:** 10.14202/vetworld.2021.1504-1510

**Published:** 2021-06-11

**Authors:** Radka Stayova Garnoeva, Mihail Dimitrov Paskalev

**Affiliations:** Department of Veterinary Surgery, Faculty of Veterinary Medicine, 6000 Stara Zagora, Bulgaria

**Keywords:** medial patellar luxation, radiography, small dog breeds, trochlear block recession, trochlear wedge recession

## Abstract

**Background and Aim:**

Anatomical and mechanical femoral angles are quite different among dog breeds. Most published data are about large dog breeds, however, medial luxation of patella is more common in small breeds. Measures of pelvic limb alignment are important for outcome of patellar luxation surgery. Therefore, the aim of the present study was to compare the values of anatomical and mechanical femoral and tibial angles in dogs before, immediately after, and 1 month after surgery for correction of medial patellar luxation (MPL).

**Materials and Methods:**

The study was conducted with 54 dogs (67 stifle joints) from four small breeds that underwent surgery by either trochlear block recession (36 stifle joints) or trochlear wedge recession (31 stifle joints) techniques.

**Results:**

In both trochleoplasty techniques, statistically significant differences in the values of the anatomical medial proximal femoral angle (aMPFA), anatomical lateral distal femoral angle (aLDFA), and femoral varus angle (in MPL Grade II) and of aMPFA and Q-angle (in MPL Grade III) were found out.

**Conclusion:**

After block recession surgery, more angles were positively influenced and this effect was more pronounced in patients with MPL Grade II.

## Introduction

In human orthopedics, reference values of anatomical and mechanical angles of extremities are routinely used to evaluate the extent of bone deformities to select the proper surgical method to restore bone conformation [1]. In veterinary patients, it should be remembered that anatomical and mechanical femoral angles are quite different among dog breeds [2,3]. Most published data are about large dog breeds [3,4], although medial luxation of patella is more common in small breeds [5].

Some investigators affirm that tibial deformities are rarely involved in the etiopathogenesis of patellar luxation and that attention should be focused on deformities of the femur [6]. The femoral varus angle (FVA) is one of most extensively studied femoral angles, reported to provide the best demonstration of femoral varus deformity [7,8]. When FVA exceeds 10° or 12°, along with aLDFA exceeding 102°, corrective osteotomy is advised [9]. In large dog breeds, normal inclination femoral angle (IFA) ranges from 140.5° to 156.5° [3], while in small breeds from 128.4° to 130.4° [10]. Reduced IFA, for example, *coxa vara* is outlined as a factor for medial patellar luxation (MPL) [11]. The quadriceps angle (Q-angle) reflects bone deformities resulting from pull force exerted by *m. quadriceps femoris*. It is changed in dogs with patellar luxation. The decreased anteversion angle, *coxa vara*, medial dislocation of *m. quadriceps femoris*, external rotation of the distal femur, internal rotation of the proximal tibia, and medial rotation of *tuberositas tibiae* are the skeletal muscle abnormalities that may lead to dislocation of the entire quadriceps mechanism and onset of patellar luxation [12]. All deformities affecting the ilium, the femur or the tibia also alter the Q-angle [13]. With respect to corrective surgery, it is also the only angle that changes post-operatively regardless of the used technique [3,12,14]. The change in aforementioned measures of pelvic limb alignment in dogs with MPL would allow for better evaluation of surgery outcome.

The present study was undertaken to compare the values of anatomical and mechanical femoral and tibial angles in dogs from small breeds before, immediately after, and 1 month after either block recession or wedge recession surgery for correction of Grades II and III MPL.

## Materials and Methods

### Ethical approval

All animals were patients of the small animal clinic at the Faculty of veterinary medicine, Stara Zagora, Bulgaria. Informed consent for participation in the study was obtained from dogs’ owners.

### Study period and location

The study was conducted from January 2015 to November 2019 in Stara Zagora, Bulgaria.

### Study cohort

Criteria for inclusion of dogs in the study comprised: (1) Small breed (only Mini-Pinscher, Chihuahua, Pomeranian, and Yorkshire Terrier), (2) type of patellar luxation (only medial), and (3) grade of MPL (only Grade II and III). Exclusion criteria were as follows: (1) Traumatic origin of the MPL and (2) any other accompanying orthopedic disease. Thus, the study was performed on 54 dogs (67 joints) from the four small breeds: 21 Mini-Pinschers, 16 Chihuahuas, 6 Yorkshire Terriers, and 11 Pomeranians without history of a previous traumatic injury. In 41 dogs, only one stifle was operated while in the other 13, both stifles underwent surgery.

All radiographic procedures in craniocaudal and mediolateral views were performed under anesthesia to ensure the proper positioning of patients during the radiography and to prevent deviations in real values of the measured angles. Dogs were first pre-medicated with 0.02 mg/kg atropine (Atropinum sulfuricum, Sopharma, Bulgaria) s.c., followed 15 min later by i.v. injection of 7.5 mg/kg tiletamine/zolazepam (Zoletil^®^ 50, Virbac, France).

Anatomical and mechanical angles of the femur and tibia were measured on digital radiographic images (iQ-VIEW/PRO version 2.7). On them, several predefined osseous landmarks were manually marked. Lines corresponding to femoral anatomical and mechanical axes and tibial mechanical axis were drawn.

**Figure-1:**
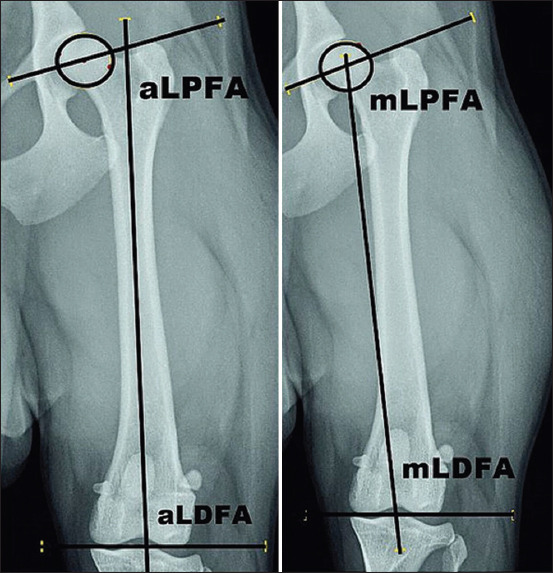
Measurement of the anatomical lateral proximal and distal femoral angles (left) and mechanical lateral proximal and distal femoral angles (right).

The following anatomical and mechanical femoral angles were measured: Anatomical lateral proximal and distal femoral angles (aLPFA and aLDFA); mechanical lateral proximal and distal femoral angles (mLPFA and mLDFA). aLPFA is formed at the intersection of the anatomical femoral axis and the line connecting the center of the femoral head and trochanter major. aLDFA is formed when the anatomical axis crosses the transcondylar axis of the femur (the line connecting most convex parts of medial and lateral femoral condyles). mLPFA is formed between the femoral mechanical axis and the line connecting femoral head center and trochanter major, whereas mLDFA is formed at the point of intersection of the femoral mechanical axis and femoral transcondylar axis (Figure-1).

**Figure-2:**
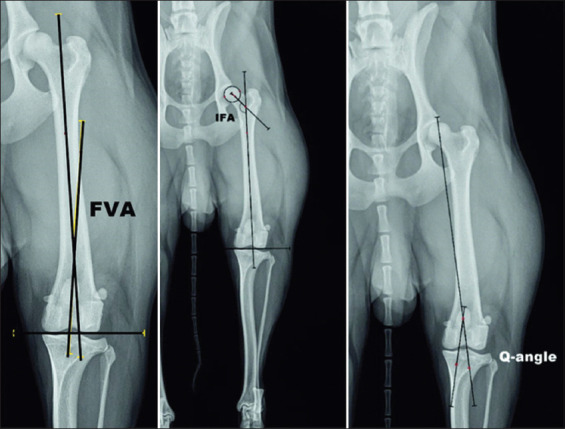
Measurement of femoral varus angle, inclination femoral angle, and quadriceps angle (Q-angle).

For measurement of IFA, the anatomical axis of femoral neck was drawn between the femoral head center and the femoral neck center. IFA is formed at the point of intersection of the two anatomical axes: That of the femur and that of the femoral neck. The FVA is formed at the intersection of anatomical femoral axis and a line, perpendicular to the transcondylar axis of the femur. The quadriceps angle (Q-angle) was measured on ventrodorsal radiographs as previously described [15]. It is formed by two lines: One passing through the origin of the rectus femoris muscle (cranial margin of the acetabulum) and the middle of the femoral trochlea, and the second – through the middle of the femoral trochlea and *tuberositas tibiae* (Figure-2).

**Figure-3:**
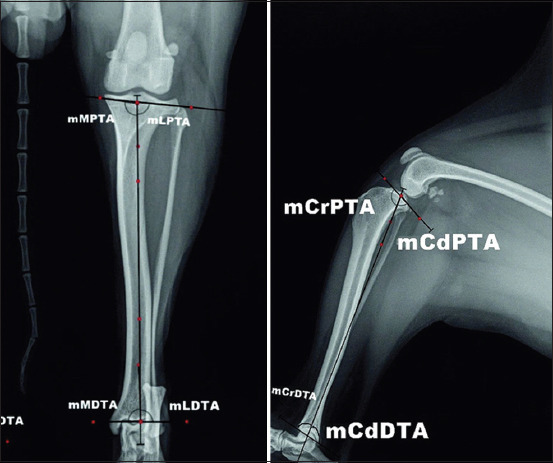
Measurement of mechanical tibial angles on radiographs in craniocaudal and mediolateral views.

Mechanical tibial angles [16,17] comprised: Mechanical medial and lateral proximal tibial angles (mMPTA and mLPTA), mechanical cranial and caudal proximal tibial angles (mCrPTA and mCdPTA); mechanical medial and lateral distal tibial angles (mMDTA and mLDTA); and mechanical cranial and caudal distal tibial angles (mCrDTA and mCdDTA). mMPTA and mLPTA were measured at the point of intersection of the mechanical axis of the tibia with the line connecting the distal points of the concavities of the medial and lateral tibial condyles. mMDTA and mLDTA are formed by the intersection of mechanical axis of the tibia with the line passing through the proximal points of the medial and lateral concavities of the tibial cochlea. mCrPTA and mCdPTA were defined by the point of intersection of the mechanical axis of the tibia and the line passing through cranial and caudal points of the tibial plateau, whereas mCrDTA and mCdDTA were measured at the point when mechanical axis of the tibia crossed the line connecting the cranial and caudal parts of distal tibial articulation surface (Figure-3).

The study included only dogs with Grade II MPL (41 joints) and Grade III MPL (26 joints). Out of them, 36 joints were submitted to block recession surgery, and 31 underwent wedge recession surgery. The joints in all dogs were closed by Mayo mattress sutures.

### Statistical analysis

The non-parametric Mann–Whitney U-test was used to evaluate the differences between healthy joints and joints affected by MPL. Values are presented as median (range). Differences were considered significant at p<0.05.

## Results

The measured femoral angles in operated dogs with Grades II and III MPL are presented in Tables-1 and 2, whereas values of measured tibial angles in the same patients – in Tables-3 and 4. The values of aLPFA and mLPFA in dogs with Grade II MPL that underwent either block recession or wedge recession surgery did not differ considerably (Table-1). aMPFA was significantly lower (71°) immediately after block recession than after wedge recession surgery (77°; p<0.05). One month after block recession surgery, this angle increased more considerably (78°), yet only slightly after wedge recession surgery (80°).

**Table-1:** Pre- and post-operative values of proximal and distal mechanical and anatomical femoral angles in stifle joints of dogs with Grade II medial patellar luxation. Values are given as median (minimum-maximum).

Angle	Surgery technique	Before surgery (n=41)	Immediately after surgery	One month after surgery
Femur proximal angles				
aLPFA	BR (n=20)	106 (91-129)	113 (94-131)	114 (91-127)
	WR (n=21)		108 (91-126)	111 (95-128)
mLPFA	BR (n=20)	105 (90-118)	110 (90-119)	114 (93-118)
	WR (n=21)		107 (90-112)	108 (90-115)
aMPFA	BR (n=20)	74 (57-95)	71 (59-92)	78 (60-94)
	WR (n=21)		77^&^ (62-89)	80 (65-90)
Femur distal angles				
aLDFA	BR (n=20)	103 (89-125)	98.5** (90-120)	97** (93-117)
	WR (n=21)		105^&^ (91-121)	101 (93-118)
mLDFA	BR (n=20)	104 (89-119)	100 (96-114)	99 (95-118)
	WR (n=21)		106 (97-116)	102 (96-114)
aMDFA	BR (n=20)	78 (68-91)	82* (72-90)	82* ((63-91)
	WR (n=21)		80 (60-89)	78 (67-90)
FVA	BR (n=20)	15 (4-33)	8.5* (2-24)	8** (2-21)
	WR (n=21)		15^&^ (6-25)	13^&&^ (6-22)
IFA	BR (n=20)	132 (117-168)	130 (114-136)	130 (123-142)
	WR (n=21)		135 (111-158)	131 (120-154)
Q-angle	BR (n=20)	22 (13-37)	18.5** (10-26)	18*** (10-29)
	WR (n=21)		19** (11-34)	18*** (10-36)

n=Number of operated joints; BR=Block recession surgery; WR=Wedge recession surgery;

* p<0.05;

** p<0.01;

*** p<0.001 versus pre-operative value;

& p<0.05;

&& p<0.01 between BR and WR for a given period. aLPFA=Anatomical lateral proximal femoral angle, aLDFA=Anatomical lateral distal femoral angle, IFA=Inclination femoral angle, FVA=Femoral varus angle

**Table-2:** Pre- and post-operative values of proximal and distal mechanical and anatomical femoral angles in stifle joints of dogs with Grade III medial patellar luxation. Values are given as median (minimum-maximum).

Angle	Surgery technique	Before surgery (n=41)	Immediately after surgery	One month after surgery
Femur proximal angles				
aLPFA	BR (n=16)	110 (93-126)	110 (92-126)	109 (93-118)
	WR (n=10)		108.5 (94-124)	113 (100-121)
mLPFA	BR (n=16)	108 (90-130)	103 (91-115)	111 (97-117)
	WR (n=10)		107.5 (92-133)	109.5 (92-114)
aMPFA	BR (n=16)	71 (59-87)	73.5 (65-97)	75.5 (62-97)
	WR (n=10)		77.5^&^ (62-97)	81.5 (56-87)
Femur distal angles				
aLDFA	BR (n=16)	107 (84-121)	101 (91-109)	99.5 (93-112)
	WR (n=10)		105 (89-119)	100 (92-110)
mLDFA	BR (n=16)	105.5 (94-119)	105 (94-109)	101.5 (94-112)
	WR (n=10)		106 (99-124)	101.5 (98-105)
aMDFA	BR (n=16)	73 (59-96]	70.5 (71-89)	80 (68-90)
	WR (n=10)		77.5 (65-90)	82.5 (78-95)
FVA	BR (n=16)	18.5 (3-34)	12* (2-19)	9** (2-23)
	WR (n=10)		15.5 (3-24)	15 (3-24)
IFA	BR (n=16)	132 (114-146)	125.5 (116-144)	129.5 (121-140)
	WR (n=10)		134 (123-144)	128.5 (126-141)
Q-angle	BR (n=16)	27 (16-44)	19** (12-35)	18** (12-32)
	WR (n=10)		24 ^&&^ (18-29)	21* (17-29)

n=Number of operated joints; BR=Block recession surgery; WR=Wedge recession surgery;

* p<0.05;

** p<0.01;

*** p<0.001 versus pre-operative value;

& p<0.05;

&& p<0.01 between BR and WR for a given period. aLPFA=Anatomical lateral proximal femoral angle, aLDFA=Anatomical lateral distal femoral angle, IFA=Inclination femoral angle, FVA=Femoral varus angle

**Table-3:** Pre- and post-operative values of proximal and distal mechanical tibial angles in stifle joints of dogs with Grade II medial patellar luxation. Values are given as median (minimum-maximum).

Angle	Surgery technique	Before surgery (n=41)	Immediately after surgery	One month after surgery
Tibia proximal angles				
mMPTA	BR (n=20)	93 (85–112)	92 (87–111)	97 (90–114)
	WR (n=21)		94 (87–108)	94 (90–108)
mLPTA	BR (n=20)	85 (72–95)	87 (70–93)	83 (70–91)
	WR (n=21)		86 (80–91)	85 (78–90)
mCrPTA	BR (n=20)	119 (90–161)	119 (112–157)	116 (112–151)
	WR (n=21)		119.5 (83–131)	118 (90–125)
mCdPTA	BR (n=20)	62 (29–79)	64.5^&^ (37–81)	66.5^&^ (41–84)
	WR (n=21)		67* (60–82)	74***(62–90)
Tibia distal angles				
mMDTA	BR (n=20)	92 (85–103)	90.5 (88–100)	90 (87–99)
	WR (n=21)		94 (87–106)	92 (90–100)
mLDTA	BR (n=20)	88 (77–101)	89 (73–94)	90 (81–93)
	WR (n=21)		87 (80–98)	90 (80–94)
mCrDTA	BR (n=20)	85 (68–105)	89.5 (72–102)	90 (70–99)
	WR (n=21)		89 (65–108)	90 (70–102)
mCdDTA	BR (n=20)	93 (75–112)	90 (79–108)	90 (74–98)
	WR (n=21)		91 (78–108)	90 (84–104)

n=Number of operated joints; BR=Block recession surgery; WR=Wedge recession surgery;

* p<0.05; *** p<0.01;

*** p<0.001 versus pre-operative value;

& p<0.05 between BR and WR for a given period.

**Table-4:** Pre- and post-operative values of proximal and distal mechanical tibial angles in stifle joints of dogs with Grade III medial patellar luxation. Values are given as median (minimum-maximum).

Angle	Surgery technique	Before surgery (n=41)	Immediately after surgery	One month after surgery
Tibia proximal angles				
mMPTA	BR (n=16)	96 (87–110)	96.5 (89–107)	96 (90–100)
	WR (n=10)		94 (88–106)	94 (92–106)
mLPTA	BR (n=16)	83.5 (72–93)	84 (73–91)	84 (80–90)
	WR (n=10)		86 (82–92)	88 (80–90)
mCrPTA	BR (n=16)	116 (106–129)	118 (109–127)	116 (110–124)
	WR (n=10)		121.5 (111–126)	118 (110–119)
mCdPTA	BR (n=16)	67 (51–88)	67 (58–74)	70 (57–75)
	WR (n=10)		69 (60–90)	72 (60–126)
Tibia distal angles				
mMDTA	BR (n=16)	92 (79–106)	91 (86–100)	90 (90–105)
	WR (n=10)		90 (85–108)	90.5 (82–106)
mLDTA	BR (n=16)	88 (78–101)	89 (80–94)	89 (75–90)
	WR (n=10)		89 (80–95)	90 (84–92)
mCrDTA	BR (n=16)	96 (72–104)	92 (72–104)	90 (74–98)
	WR (n=10)		86.5 (71–103)	83 (78–100)
mCdDTA	BR (n=16)	86 (75–108)	89 (72–108)	90 (3–100)
	WR (n=10)		88.5 (82–109)	91 (82–98)

n=Number of operated joints; BR=Block recession surgery; WR=Wedge recession surgery

Surgical correction resulted in statistically significant difference in aLDFA: 98.5° immediately after block recession versus wedge recession surgery – 105° (p<0.05). One month later, aLDFA decreased to 97° in joints that underwent block recession surgery (p<0.05).

Post-operative values of FVA after block recession were lower (8.5°) than after wedge recession (15°; p<0.05). One month later, the FVA after block recession decreased even more (8°), while after wedge recession, its value was close to pre-operative value (13°). The differences in FVA after both surgical techniques were statistically significant (p<0.01). The FVA was higher before block recession surgery (15°) compared to its post-operative value (8.5°; p<0.05). Such tendency was not observed with the other surgical technique. The differences in FVA by the 1^st^ post-operative month were even more pronounced (8° vs. 13°; p<0.01).

Q-angle was significantly changed in joints with Grade II MPL before, immediately after, and 1 month after surgery using both operative approaches. After block recession surgery, the Q-angle decreased significantly from 22° to 18.5° (p<0.05) and was even lower by the 1^st^ post-operative month (18°; p<0.001). The same trend was observed for the other surgical technique. The Q-angle was identical 1 month after application of both surgical techniques (18°).

In dogs with Grade III MPL (Table-2), aMPFA was statistically significantly lower (p<0.05) after block recession (73.5°) compared to wedge recession surgery (77.5°). Block recession surgery resulted in a significant decrease of FVA from 18.5° to 12° (p<0.05) and 1 month later to 9° (p<0.01), whereas wedge recession surgery did not change its pre-operative values. Q-angle decreased after surgery by both block and wedge recession to 19° and 24°, respectively (p<0.01). It was even lower than pre-operative values 1 month after both block recession (18°; p<0.01) and wedge recession surgery (21°; p<0.05).

Only one of the measured tibial angles showed statistically significant differences between both surgical techniques immediately after the surgery and 1 month later – mCdPTA in dogs with Grade II MPL (Table-3). This angle increased in joints following wedge recession surgery (67°) compared to those with block recession surgery (64.5°; p<0.05). The same trend was preserved 1 month later as well: 74° vs. 66.5°, respectively (p<0.05). Wedge recession surgery was found to increase significantly the pre-operative mCdPTA value (from 62° to 74°; p<0.001).

## Discussion

Femoral and tibial deformities accompany medial patellar luxation; out of them, femoral is more pronounced and of greater clinical relevance. Varus deformity of the distal femur is exceptionally important in MPL pathogenesis [2,4,-18-20]. Therefore, operative treatment should be aimed at correction of the distal femur [2,21]. A lot regaining normal values [14]. The values of FVA and aLDFA determine distal femur varus [14]. With this regard, some authors recommend corrective osteotomy of the femur when FVA is greater than 12° and aLDFA is greater than 102° [22,23]. This approach brings back FVA to its reference values or to zero, whereas aLDFA equals 90° [4,22]. The applied wedge recession surgery did not result in FVA reduction in patients with both grades of MPL, whereas this occurred after block recession of the trochlea with statistically significant difference versus pre-operative values. Only in Grade II MPL, FVA differed between both used techniques. The same tendency was found out for the other two angles associated to skeletal deformities, for example, aLDFA and mLDFA. They decreased and approached reference values after block recession surgery [10] confirming that correction of bone deformities was better with this technique. As wedge recession was concerned, angles determining varus deformity (FVA and aLDFA) decreased although insignificantly. Post-operative FVA and aLDFA values were comparable to those measured in healthy joints of dogs from small breeds but only when the technique was used in joints with Grade II MPL [7,10]. Therefore, wedge recession of the trochlea with lateral imbrication of the joint capsule and *fascia lata* may be applied in Grade II MPL but is not recommended for Grade III MPL due to more severe bone deformities.

The greater the post-operative aLDFA, the higher the probability for complications or poor outcome of surgery [24]. In our study, aLDFA values decreased after application of both methods, yet with block recession surgery, results were better and angles were closer to values of healthy joints.

The main angle that decreases during the post-operative period regardless of the used surgical technique is the Q-angle [5,25]. The patella, together with *m. quadriceps femoris*, is part of the knee extensor mechanism [13]. Thus, deviations in one or more elements of this mechanism result in changes in the quadriceps angle too. The force generated by quadriceps muscle leads to axial deviation of 10° medially; therefore, this value is referred to as normal [13]. In our study, all pre-operative values of the Q-angle were higher.

Miles *et al*. [26] observed a positive correlation between the MPL grade and the Q-angle. In our study, Q-angle was higher in joints with Grade III (27°) compared to Grade II MPL (22°). Immediately after both used surgical techniques, the values of the angle decreased and were close to those reported by Pinna and Romagnoli [15] as the patella and the insertion of *m. quadriceps femoris* regained their normal position.

Tibial deformities are less important for MPL etiopathogenesis and development [27]. In the present study, statistically significant changes were observed only in mechanical caudal proximal tibial angle (mCdPTA) in dogs with Grade II MPL. This angle, which is not associated with patellar luxation, increased after application of both operative interventions, although statistically significantly only immediately after and 1 month after wedge recession surgery. It should be noted that mCdPTA increase was reported to increase the risk from rupture of the cranial cruciate ligament [28] – a fact in favor of block recession as a method of choice in MPL treatment. None of other tibial angles in dogs with MPL Grades II and III were significantly altered postoperatively.

## Conclusion

The results of this study allowed concluding that surgical treatment of medial patellar luxation in dogs from small breeds using two trochleoplasty methods resulted in improved values of aMPFA, aLDFA, and FVA (in Grade II) and of aMPFA and Q-angle (in Grade III luxation). The comparison to pre-operative values demonstrated that more angles were positively influenced after application of trochlear block recession, particularly in Grade II MPL patients. The progressive improvement of radiological measures of limb alignment by the 1^st^ post-operative month compared to the immediate post-operative period achieved with both techniques without corrective osteotomy suggested that observed bone deformities were rather a consequence than a cause for the onset of medial patellar luxation.

## Authors’ Contributions

RSG: Conception and design of the study. RSG and MDP: All surgical interventions. RSG and MDP: Radiography, acquisition, statistical analysis, and interpretation of the data. RSG: Composition and revision of the manuscript. Both authors read and approved the final version of the manuscript.
